# The Prioritization of Clinical Risk Factors of Obstructive Sleep Apnea Severity Using Fuzzy Analytic Hierarchy Process

**DOI:** 10.1155/2015/257856

**Published:** 2015-06-15

**Authors:** Thaya Maranate, Adisak Pongpullponsak, Pimon Ruttanaumpawan

**Affiliations:** ^1^Department of Mathematics, King Mongkut's University of Technology Thonburi, Bangkok 10140, Thailand; ^2^Department of Medicine, Faculty of Medicine Siriraj Hospital, Mahidol University, Bangkok 10700, Thailand

## Abstract

Recently, there has been a problem of shortage of sleep laboratories that can accommodate the patients in a timely manner. Delayed diagnosis and treatment may lead to worse outcomes particularly in patients with severe obstructive sleep apnea (OSA). For this reason, the prioritization in polysomnography (PSG) queueing should be endorsed based on disease severity. To date, there have been conflicting data whether clinical information can predict OSA severity. The 1,042 suspected OSA patients underwent diagnostic PSG study at Siriraj Sleep Center during 2010-2011. A total of 113 variables were obtained from sleep questionnaires and anthropometric measurements. The 19 groups of clinical risk factors consisting of 42 variables were categorized into each OSA severity. This study aimed to array these factors by employing Fuzzy Analytic Hierarchy Process approach based on normalized weight vector. The results revealed that the first rank of clinical risk factors in Severe, Moderate, Mild, and No OSA was nighttime symptoms. The overall sensitivity/specificity of the approach to these groups was 92.32%/91.76%, 89.52%/88.18%, 91.08%/84.58%, and 96.49%/81.23%, respectively. We propose that the urgent PSG appointment should include clinical risk factors of Severe OSA group. In addition, the screening for Mild from No OSA patients in sleep center setting using symptoms during sleep is also recommended (sensitivity = 87.12% and specificity = 72.22%).

## 1. Introduction

Obstructive sleep apnea (OSA) is a common medical disorder characterized by repetitive partial or complete collapse of the upper airway during sleep, resulting in sleep fragmentation and cyclic oxygen desaturation. The prevalence of OSA in adult population is approximately 3–7% for males and 2–5% for females [1–4]. This can be as high as 50–98% in the morbidly obese population [5]. OSA leads to neurocognitive consequences for example, unrefreshing sleep, excessive daytime sleepiness, motor vehicle accidents and work performance [6], impaired quality of life, and considerable morbidity for cardiovascular diseases [7, 8] as well as a substantial economic impact [9]. OSA can progress to more severe if it is left untreated. Diagnosis and severity assessment required Respiratory Disturbance Index (RDI) obtained from polysomnography (PSG). The RDI is defined as the total numbers of apneas, hypopneas, and respiratory-effort related arousals (RERAs) per hour of sleep (events/hour).

Unfortunately, PSG is not widely available in Thailand because of the relative lack of sleep laboratories and results in a very long waiting list. Delayed diagnosis and treatment may lead to worse outcomes particularly in patients with Severe OSA. Therefore, the appointment for PSG should be based on OSA severity rather than the first-come-first-serve basis. Moreover, clinicians prefer a simple and nonexpensive tool to predict the severity of OSA. To date, no single clinical information can predict the severity of OSA. We believe that if symptoms and anthropometric data are categorized into groups, this may solve the problem. We previously studied 113 variables of 1,042 sleep questionnaires, anthropometric measurements, and PSGs data from suspected OSA patients of Siriraj Sleep Center. Using factor analysis, the 19 groups of clinical risk factors consisting of 42 variables were categorized into each OSA severity [10]. This research is the extended study aiming to prioritize these 19 groups of factors in each level of OSA severity by using Fuzzy Analytic Hierarchy Process (FAHP) approach. The study has been approved by the Ethics Committee of Siriraj Institutional Review Board, Faculty of Medicine Siriraj Hospital, Mahidol University, Thailand.

## 2. Materials and Methods

### 2.1. Patients, Sleep Questionnaire, and Polysomnography

The 1,042 suspected OSA patients who underwent diagnostic PSGs at Siriraj Sleep Center, Siriraj Hospital, during 2010-2011 were studied. Sleep questionnaires and anthropometric measurements were obtained in all patients in the evening prior to undergoing PSG. Self-administered sleep questionnaire consisted of 5 domains (113 questions), including (1) patients' characteristics; (2) work and sleep pattern; (3) nighttime symptoms; (4) daytime symptoms, and (5) Epworth Sleepiness Scale. Anthropometric measurements including body weight, height, neck, waist, and hip circumferences, and thyromental distance were measured by sleep technicians. All patients underwent PSGs using standard acquisition techniques. All PSGs were manually scored by certified advanced sleep technicians and reviewed by certified sleep specialists using standard scoring rule AASM 2007 [16]. Patients with central sleep apnea were excluded. Patients with RDI of <5.0, 5.0–14.9, 15.0–30.0, and >30 events/hour are classified as having No OSA, Mild OSA, Moderate OSA, and Severe OSA, respectively. The distribution of OSA severity is shown in Table 1.

### 2.2. The Hierarchical Structure of 19 Clinical Risk Factors of All OSA Severities from Factor Analysis [10]

**Figure 1:**
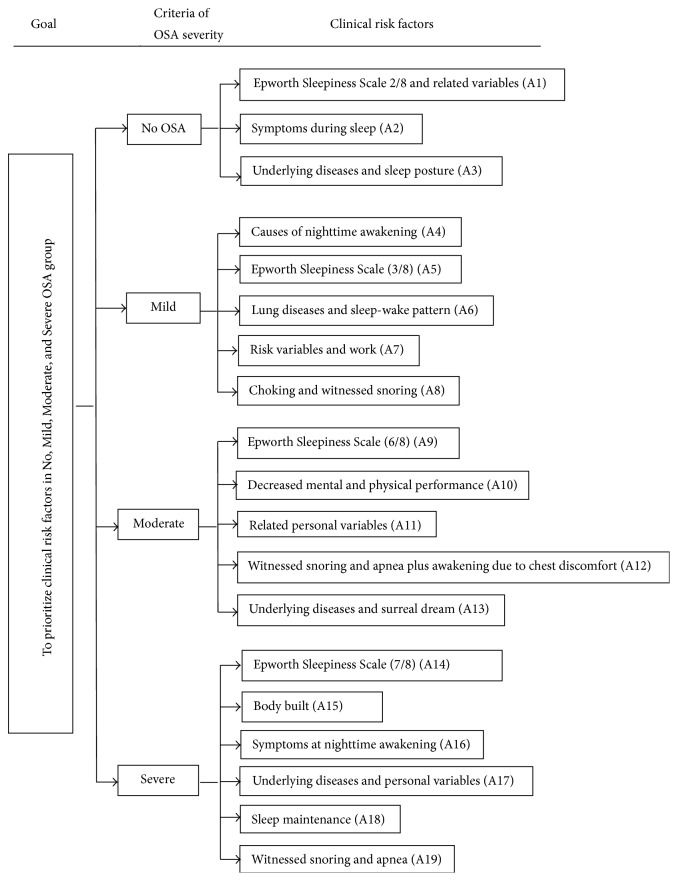
The preprioritized hierarchical structure of 19 clinical risk factors of all OSA severities.

The abovementioned 42 variables which were categorized into No, Mild, Moderate, and Severe OSA group comprised 3, 5, 5, and 6 clinical risk factors, respectively. All these factors will be prioritized according to their importance to OSA (Figure 1).

### 2.3. A Questionnaire of Analytic Hierarchy Process (AHP)

We created questionnaire of AHP using 9-point scale [11–15] (Table 2). The pairwise comparisons between clinical risk factors were performed within each level of OSA severity. Therefore, there were 3, 10, 10, and 15 comparative questions in No, Mild, Moderate, and Severe OSA, respectively.

### 2.4. A Specialist Team

The questionnaire of AHP was taken to 3 sleep specialists. They were individually face-to-face interviewed by author to determine which groups of factors they thought to be more important when compared to another. Then, the decision data were collected.

### 2.5. The Construction and Consistency Check of Pairwise Comparison Matrix [17]

**Step 1 (establishing the hierarchical structure (Figure 1)).**

Then, the decision-makers are requested to make pairwise comparisons between decision alternatives and criteria using a nine-point scale from Table 2. Subsequently, all matrices are developed and all pairwise comparisons will be obtained from each *n* decision-maker.

**Step 2 (calculating the consistency).**

To ensure that the priority of elements is consistent, the maximum eigenvector or relative weights and *λ*_max_ are calculated. Then, the consistency index (CI) for each matrix order *n* using (1) is computed. Based on the CI and random index (RI), the consistency ratio (CR) is calculated by (2):

(1) CI=λmax−nn−1,

(2) CR=CIRI,

where RI is the random consistency index obtained from a randomly generated pairwise comparison matrix.  Table 3 shows the values of the RI for matrices of orders 1 to 15 [12]. If the value of CR is 0.1 or less, the pairwise comparisons will be considered as having an acceptable consistency.

Then, we construct a fuzzy pairwise comparison matrix in each criterion.

### 2.6. The Mathematics of Fuzzy Sets and Triangular Fuzzy Number (TFN) [18]

The fuzzy set theory is an effective instrument for modeling in the lack of comprehensive and accurate information. A TFN is a particular fuzzy set $\tilde{C}$, and its membership function $\mu_{\tilde{C}}(x)$ is a continuous linear function. A TFN is defined by its basic particular equation which is [19]

(3) $$\begin{array}{c} \mu_{\tilde{C}}\left(\;x\;\right)=\left\{\begin{array}{cc} 0, & x<1,\\ \frac{\left(x-l\right)}{\left(m-l\right)}, & l\leq x<m,\\ 1, & x=m,\\ \frac{\left(u-x\right)}{\left(u-m\right)}, & m<x\leq u,\\ 0, & u<x, \end{array}\right. \end{array}$$

where *l* and *u* correspond to the lower and upper bounds of the fuzzy number $\tilde{C}$, respectively, and *m* is the midpoint. A TFN is indicated as $\tilde{C}=(l,m,u)$. Arithmetic operations between fuzzy numbers or a fuzzy number and crisp number have been defined elsewhere in Bulut and Zadeh [18, 19] by standard fuzzy arithmetic operations.

In this research, we use TFN to prioritize clinical risk factors of No, Mild, Moderate, and Severe OSA groups with fuzziness. A TFN is designated as *M*_*ij*_ = (*l*_*ij*_, *m*_*ij*_, *u*_*ij*_).

Considering two TFNs, let *M*_1_ = (*l*_1_, *m*_1_, *u*_1_) and *M*_2_ = (*l*_2_, *m*_2_, *u*_2_). Their operation laws are as follows [20]:

(4) l1,m1,u1⊕l2,m2,u2=l1+l2,m1+m2,u1+u2,l1,m1,u1⊗l2,m2,u2=l1×l2,m1×m2,u1×u2,l1,m1,u1−1=1u1,1m1,1l2.

### 2.7. The Construction of Fuzzy Pairwise Comparison Matrix

Consider the following:

(5) $$\begin{array}{c} \begin{array}{ccccc} FACTOR & C_{1} & C_{2} & \cdots & C_{M}\\ \begin{array}{c} C_{1}\\ C_{2}\\ \vdots \\ C_{M} \end{array} & \left[\begin{array}{c} 1\\ c_{21k}\\ \vdots \\ c_{M1k} \end{array}\right. & \begin{array}{c} c_{12k}\\ 1\\ \vdots \\ c_{M2k} \end{array} & \begin{array}{c} \cdots \\ \cdots \\ \cdots \\ \cdots \end{array} & \left.\begin{array}{c} c_{1Mk}\\ c_{2Mk}\\ \vdots \\ 1 \end{array}\right] \end{array}, \end{array}$$

where VM_1_, PM_2_, and NM_3_ are pairwise comparison matrix of each decision-maker *k* and *c*_*ijk*_ is the pairwise comparison score of each decision-maker *k*. Integrating 3 decision-makers' grades through (6) and *M*_*gi*_^*j*^ yields TFN:

(6) Mgij=lij,mij,uij  such  aslij=mink⁡cijk,  mij=1K∑k=1Kcijk,  uij=maxk⁡cijk.

By this procedure, decision-makers' pairwise comparison values are transformed into TFN. After forming fuzzy pairwise comparison matrix, weights of all factors are determined by FAHP method.

In this study, the extent FAHP which was originally introduced by Chang [20] is utilized. Let *X* = {*x*_1_, *x*_2_, *x*_3_,…, *x*_*n*_} be an object set and *G* = {*g*_1_, *g*_2_, *g*_3_,…, *g*_*n*_} a decision set. According to Chang's extent analysis, each decision is taken and extent analysis for each goal is performed, respectively. Therefore, *m* extent analysis values for each decision can be obtained with the following:

(7) $$\begin{array}{c} signs\;\;\left[\begin{array}{cccc} M_{g1}^{1} & M_{g1}^{2} & \cdots & M_{g1}^{m}\\ M_{g2}^{1} & M_{g2}^{2} & \cdots & M_{g2}^{m}\\ \vdots & \vdots & \vdots & \vdots \\ M_{gn}^{1} & M_{gn}^{2} & \cdots & M_{gn}^{m} \end{array}\right]. \end{array}$$

If *i* = *j* then *M*_*gi*_^*j*^ = (1, 1, 1).

### 2.8. Procedure of FAHP [21–27]

Chang's extent analysis can be performed in the following steps.

**Step 1 .**

The value of fuzzy synthetic extent with respect to the *i* decision is defined as

(8) Si=∑i=1mMgij⊗∑i=1n∑j=1mMgij−1,∑j=1mMgij=∑j=1mlj,∑j=1mmj,∑j=1muj,∑i=1n∑j=1mMgij=∑i=1nli,∑i=1nmi,∑i=1nui,∑i=1n∑j=1mMgij−1=1∑i=1nui,1∑i=1nmi,1∑i=1nli.

We calculate fuzzy criteria weights of TFN of *S*_*i*_ = (*l*_*i*_, *m*_*i*_, *u*_*i*_) by (8).

**Step 2 .**

Let *M*_1_ = (*l*_*l*_, *m*_*l*_, *u*_*l*_) and *M*_2_ = (*l*_2_, *m*_2_, *u*_2_) be 2 TFNs; the degree of possibility of *M*_2_ ≥ *M*_*l*_ is defined as

(9) $$\begin{array}{c} V\left(\;M_{2}\geq M_{1}\;\right)=\underset{y\geq x}{\sup}\left\lfloor \;\min\left(\;\mu_{M_{1}}\left(x\right),\mu_{M_{2}}\left(y\right)\;\right)\;\right\rfloor . \end{array}$$

Since the height of a fuzzy set hgt (*C*) is the supremum (maximum) of the membership grades of *C*, therefore,

(10) VM2≥M1=hgtM1∩M2=μM2d=1if  m2>m10if  l1>u2l1−u2m2−u2−m1−l1otherwise.

**Step 3 .**

The degree of possibility for a convex fuzzy number to be greater than *k* convex fuzzy *M*_*i*_  (*i* = 1, 2, …, *k*) numbers can be defined by

(11) VM≥M1,M2,…,Mk=VM≥M1,M≥M2,…,M≥Mk=min⁡VM≥Mii=1,2,…,k.

Assume that *d*′(*C*_*i*_) = min⁡*V*(*S*_*i*_ ≥ *S*_*k*_) for *k* = 1, 2, …, *n*; *k* ≠ *i*; then the weight vector is given by *w*′ = (*d*′(*C*_1_), *d*′(*C*_2_),…, *d*′(*C*_*n*_))^T^ where *C*_*i*_  (*i* = 1, 2, …, *n*) are *n* elements.

**Step 4 .**

Via normalization, the normalized weight vectors are

(12) $$\begin{array}{c} w={\left(\;d\left(\;C_{1}\;\right),d\left(\;C_{2}\;\right),\ldots ,d\left(\;C_{n}\;\right)\;\right)}^{T}, \end{array}$$

where *w* is a nonfuzzy number. Then, weights of main criteria and attributes (*w*_*i*_) can be calculated by

(13) $$\begin{array}{c} w_{i}=\frac{w_{i}^{\prime }}{\sum_{i=1}^{n}w_{i}^{\prime }}. \end{array}$$

### 2.9. Diagnostic Test Evaluation of Sensitivity, Specificity, and 95% Confidence Interval (95% CI) [28, 29]

Sensitivity and specificity are statistical measures of the performance of a binary classification test. Sensitivity is the proportion of people with the target disorder in whom the test result is positive. Specificity is the proportion of people without the target disorder in whom test result is negative. To use these concepts, we divide test results into normal and abnormal to create a 2 × 2 table (Table 4):

(14) Sensitivityaa+b=atest  positivea+btest  positive+false  negative,Specificitydc+d=dtrue  negativec+dfalse  positive+true  negative.

The 95% CI of a proportion is estimated based on the binomial theorem:

(15) $$\begin{array}{c} p\pm 2\sqrt{\frac{p\left(1-p\right)}{N}}, \end{array}$$

where *p* is the observed proportion and *N* is the number of people observed.

### 2.10. The Summarization of All Steps for the Prioritization of Clinical Risk Factors

See (Figure 2).

## 3. Results

### 3.1. Decision-Makers' Data

Questionnaires of AHP in No OSA and OSA group were taken to the 3 decision-makers, VM_1_, PM_2_, and NM_3_, to weight according to the significance to OSA. Main criteria of pairwise comparison matrices of No OSA and OSA groups by 3 sleep specialists (VM_1_, PM_2_, and NM_3_) are as follows. A1–A19 are clinical risk factors in each OSA severity.

No OSA

(16) VM1=A1A2A3115451814181PM2=A1A2A3117371813181NM3=A1A2A31451415415451

Mild OSA

(17) VM1=A4A5A6A7A81343161311211614211215131211666561PM2=A4A5A6A7A811412151741121517221141755411477741NM3=A4A5A6A7A8111321132411313213121312121412121

Moderate OSA

(18) VM1=A9A10A11A12A1311561435181251618117242718131512181PM2=A9A10A11A12A1314514314121713151211713477171333171NM3=A9A10A11A12A13113151114132131411613536151123121

Severe OSA

(19) $$\begin{array}{c} {\text{VM}}_{1}=\begin{array}{cccccc} A14 & A15 & A16 & A17 & A18 & A19\\ 1 & 3 & \frac{1}{3} & 2 & \frac{1}{3} & \frac{1}{5}\\ \frac{1}{3} & 1 & \frac{1}{4} & \frac{3}{7} & \frac{1}{5} & \frac{1}{7}\\ 3 & 4 & 1 & 2 & \frac{1}{2} & \frac{1}{2}\\ \frac{1}{2} & \frac{7}{3} & \frac{1}{2} & 1 & 2 & \frac{1}{3}\\ 3 & 5 & 2 & \frac{1}{2} & 1 & \frac{1}{2}\\ 5 & 7 & 2 & 3 & 2 & 1 \end{array}\\ {\text{PM}}_{2}=\begin{array}{cccccc} A14 & A15 & A16 & A17 & A18 & A19\\ 1 & \frac{1}{4} & \frac{1}{7} & \frac{1}{3} & 4 & \frac{1}{6}\\ 4 & 1 & \frac{1}{2} & 4 & 4 & \frac{1}{5}\\ 7 & 2 & 1 & 4 & 6 & \frac{1}{2}\\ 3 & \frac{1}{4} & \frac{1}{4} & 1 & 3 & \frac{1}{7}\\ \frac{1}{4} & \frac{1}{4} & \frac{1}{6} & \frac{1}{3} & 1 & \frac{1}{7}\\ 6 & 5 & 2 & 7 & 7 & 1 \end{array}\\ {\text{NM}}_{3}=\begin{array}{cccccc} A14 & A15 & A16 & A17 & A18 & A19\\ 1 & 4 & \frac{1}{5} & 2 & \frac{1}{2} & \frac{1}{7}\\ \frac{1}{4} & 1 & \frac{1}{4} & \frac{1}{2} & \frac{1}{2} & \frac{1}{5}\\ 5 & 4 & 1 & 4 & 3 & \frac{1}{5}\\ \frac{1}{2} & 2 & \frac{1}{4} & 1 & 1 & \frac{1}{5}\\ 2 & 2 & \frac{1}{3} & 1 & 1 & \frac{1}{5}\\ 7 & 5 & 5 & 5 & 5 & 1 \end{array} \end{array}$$

### 3.2. Results of Consistency Test

Consistency ratio for each specialist's decision was calculated and checked. The results revealed that all CR values were ≤0.1. Thus, the consistency of all the decisions were satisfactory (Table 5).

From Table 5, the decision comparison matrices of the 3 sleep specialists in each severity group are then transformed into TFN by using (6) (Tables 6 [Table tab7] [Table tab8]–9).

### 3.3. Procedure of Fuzzy Analytic Hierarchy Process (FAHP)

*Step 1*. We employ the calculation of fuzzy synthetic extents with respect to factors where the results of *S*_A1_–*S*_A19_ are calculated in detail (Table 10).

From the calculation, the weights of significance of decision factors in terms of triangle fuzzy number with lower, mean, and upper bounds (*l*_*i*_, *m*_*i*_, *u*_*i*_) in each OSA severity are obtained.

*Step 2*. We compare the values of *S*_*i*_, respectively, and calculate the degree of possibility of *S*_*j*_ = (*l*_*j*_, *m*_*j*_, *u*_*j*_) ≥ *S*_*i*_ = (*l*_*i*_, *m*_*i*_, *u*_*i*_), yielding both values of *V*(*S*_*j*_ ≥ *S*_*i*_) and *V*(*S*_*i*_ ≥ *S*_*j*_) in all groups (Tables 11 [Table tab12] [Table tab13]–14).

*Step 3*. The minimum degree of possibilities values of clinical risk factors in No OSA to Severe OSA are calculated by (11) as in Table 15.

*Step 3 to Step 4*. We obtain *w*′ = (*d*′(*C*_1_), *d*′(*C*_2_),…, *d*′(*C*_*n*_))^T^ and *w* = (*d*(*C*_1_), *d*(*C*_2_),…,*d*(*C*_*n*_))^T^ of all criteria where *w* is a nonfuzzy number as shown in Table 16.

At this stage, factors affecting each level of OSA severity have been prioritized by using the FAHP methodology, which is a scientific procedure of multicriteria decision-making method. It can reflect effectively the human thoughts with vagueness of real world decision-making. The results of this research have finally provided the optimal factors.

### 3.4. Final Ranking, Choosing the Optimal Factors Sensitivity/Specificity and 95% CI

Next, we put them in an order from highest to lowest based on what the priority weight of each factor is and on their corresponding normalized weights vector. An optimal factor that has the highest score in a priority rating is selected. Finally, the overall sensitivity, specificity, and 95% CI of all OSA severities are calculated (Table 17).

As can be seen in Table 17, in No OSA, first rank clinical risk factor is symptoms during sleep and the last one is underlying diseases and sleep posture. In Mild OSA, first rank clinical risk factor is choking and witnessed snoring and the last one is lung diseases and sleep-wake pattern. In Moderate OSA, first rank clinical risk factor is witnessed snoring and apnea plus awakening due to chest discomfort, whereas the last one is related personal variables. In Severe OSA, first rank clinical risk factor is witnessed snoring and apnea and the last one is underlying diseases and personal variables. It is observed that the sensibility and specificity of the approach to each group are high.

## 4. Discussion

Regarding the concept of factor analysis, each factor has its members (variable or symptom); whenever any variable is found, there is high tendency of the rest members to occur because they belong to the same factor. The FAHP methodology can provide the flexibility and robustness needed for the decision-maker to understand the decision problem as well as a standard control of consistency on the decision matrix for them. These merits of the approach lead to the developed FAHP questionnaire for detecting OSA patient in each level.

In Severe OSA group, the most related clinical risk factor is witnessed snoring and apnea that includes the witnessing of frequency of periodically stopped breathing and snoring as well as the intensity of the loudness of snoring. From Table 1 the Severe OSA patients have very high RDI (range 30.1–168.2 events/hour and mean ± S.D. = 60.6 ± 25.3 events/hour). Thus, in clinical practice, we propose that the appointment for the urgent sleep study and prompt management should include all clinical risk factors of Severe group (the sensitivity of 92.32% and specificity of 91.76%) (Table 18).

In Moderate OSA, the overall sensitivity and specificity of the approach are 89.52% and 88.18%. Furthermore, the most at-risk factor is witnessed snoring and apnea plus chest discomfort as a cause of awakening during late night or getting up earlier than expectation. It should be noteworthy that apnea and snoring are observed in this Moderate group as well as in Severe group. Among 5 groups of factors in Moderate OSA, the first rank group carries extremely high normalized weight vector (0.481) compared to the remaining 4 groups (0–0.291). Therefore, in addition to the severe group the first rank clinical risk factors of Moderate OSA may be included in the urgent PSG appointment.

For the remaining clinical risk factors and variables in Moderate, Mild, and No OSA groups, they should be indicated for queueing up in the usual PSG waiting list.

In No OSA group, the details of symptoms during sleep included the troubles at night or during sleep within the last month, that is, someone's notice (witness) of having periodically grasping hands and profound sweating during sleep. The frequencies of these symptoms are positively related to RDI [10]. The second running up clinical risk factor is Epworth Sleepiness Scale (2/8) and related variables. Epworth Sleepiness Scale (2/8), which is negatively related to RDI, still has no excessive daytime sleepiness even in sleeping induced atmosphere, that is, being a passenger in a car for an hour without break and sitting inactive in a public place. This is obvious evidence to separate No OSA group from Mild OSA (with positively correlated with RDI). The related variables with positive correlation to RDI are age, hypertension, and routine medicine use. Therefore, the elderly with hypertension and medicine routine use having no excessive daytime sleepiness, though having symptoms during sleep, should be really taken into account in No OSA group (sensitivity = 96.49%, specificity = 81.23%).

However, in the prioritization of Mild and No OSA group, the symptom during sleep in No OSA is at the borderline adjacent to Mild OSA. Herein, we propose that the screening for Mild from No OSA patients in sleep center setting may be implemented with the questionnaire which covers first rank clinical risk factor of No OSA group, that is, symptoms during sleep within the last month with the sensitivity of 87.12% and specificity of 72.22% (Table 19).

To date, there have been no known previous studies concerning sleep questionnaire and anthropometrics data as the clinical risk factors for the prioritization of PSG appointment based on OSA severity.

Our future work will be planning to create the formula of clinical information for urgent sleep study appointment and screening of Mild OSA using fuzzy binary logistic regression equation approach.

## 5. Conclusion

Using FAHP based on normalized weight vectors to prioritize 19 clinical factors revealed that in each severity group based on RDI as No, Mild, Moderate, and Severe OSA, their first clinical risk factors are nighttime symptoms. Then, the prioritized factors are selected to propose the criteria for sleep study appointment. Therefore, the urgent sleep study appointments based on clinical risk factors of Severe OSA have been presented. In addition, the screening for Mild from No OSA patients in sleep center setting using symptoms during sleep is recommended. Finally, the questionnaires for these purposes can be constructed to cover the concerning factors.

## Figures and Tables

**Figure 2:**
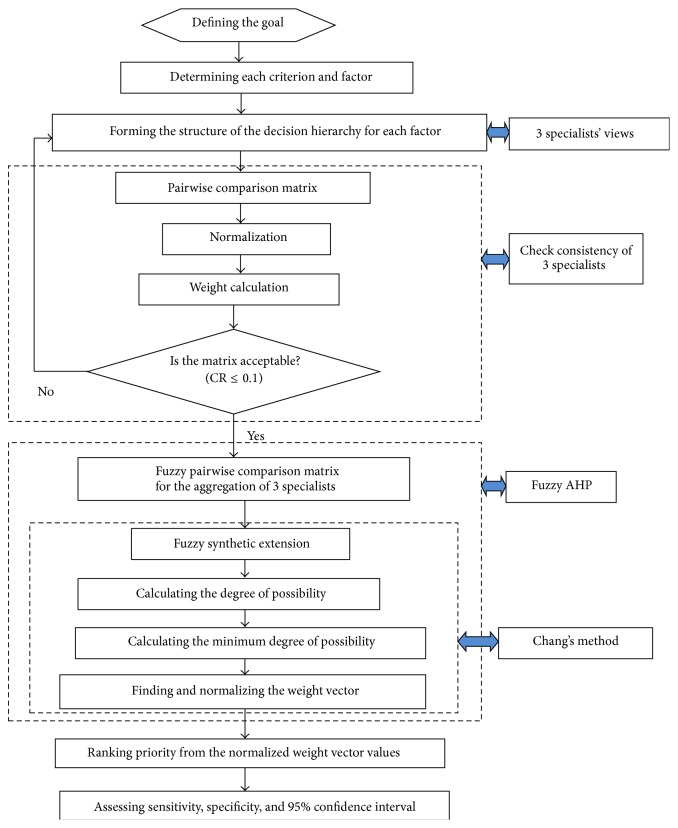
Flow chart for the prioritization of clinical risk factors.

**Table 1:** Patients' characteristics.

OSA severity	Number of patients	Age (years)	Sex		RDI (events/hour)
		Range (mean ± SD)	Male (%)	Female (%)	Range (mean ± SD)
No OSA	62	23–85 (49.6 ± 16.1)	38.7	61.3	0.0–4.9 (2.9 ± 1.3)
Mild OSA	178	20–90 (50.6 ± 13.3)	45.8	54.2	5.0–14.9 (10.1 ± 3.0)
Moderate OSA	262	19–83 (54.7 ± 13.3)	55.9	44.1	15.0–29.8 (22.0 ± 4.3)
Severe OSA	540	19–88 (54.8 ± 12.8)	69.6	30.4	30.1–168.2 (60.6 ± 25.3)

**Table 2:** 9-point intensity of relative weight (importance or well-being) scale (adapted from [11–15]).

Intensity of importance/well-being	Definition	Significance
1	Equal importance/equally good	Two activities contribute equally to objective

3	Moderate importance of one factor over another/weakly	Experience and judgment slightly favor one activity over another

5	Strong or essential importance/strongly	Experience and judgment strongly favor one activity over another

7	Very strong importance/very strongly	An activity is strongly favored, and its dominance is demonstrated in practice

9	Extreme importance/absolutely better	The evidence favoring one activity over another is of the highest possible order of affirmation

2,4, 6,8	Intermediate value between the two adjacent judgments	When a compromise is needed

Reciprocals of the above nonzero numbers	Reciprocals for inverse comparison	

**Table 3:** Random index (RI) [12].

*N*	1	2	3	4	5	6	7	8	9	10	11	12	13	14	15

RI	0.00	0.00	0.58	0.90	1.12	1.24	1.32	1.41	1.45	1.49	1.51	1.48	1.56	1.57	1.58

**Table 4:** The relation between a diagnostic test result and occurrence of disease.

		Disease	
		Present	Absent
Test	Positive	*a* = test positive	*c* = false positive
	Negative	*b* = false negative	*d* = true negative

**Table 5:** Consistency test for each sleep specialist's decision.

Criteria	Factors	Consistency ratio			Consistency test
		1	2	3	
Severe OSA	Epworth Sleepiness Scale (7/8) (A14) Body built (A15) Symptoms at nighttime awakening (A16) Underlying diseases and personal variables (A17) Sleep maintenance (A18) Witnessed snoring and apnea (A19)	0.099	0.095	0.097	Accepted

Moderate OSA	Epworth Sleepiness Scale (6/8) (A9) Decreased mental and physical performance (A10) Related personal variables (A11) Witnessed snoring and apnea plus awakening due to chest discomfort (A12) Underlying diseases and surreal dream (A13)	0.096	0.062	0.029	Accepted

Mild OSA	Causes of nighttime awakening (A4) Epworth Sleepiness Scale (3/8) (A5) Lung diseases and sleep-wake pattern (A6) Risk variables and work (A7) Choking and witnessed snoring (A8)	0.085	0.099	0.061	Accepted

No OSA	Epworth Sleepiness Scale (2/8) and related variables (A1) Symptoms during sleep (A2) Underlying diseases and sleep posture (A3)	0.083	0.093	0.056	Accepted

**Table 6:** Fuzzy pairwise comparison matrix for the clinical risk factors A1–A3 in No OSA group in terms of TFN.

Factors	A1	A2	A3
A1	(1, 1, 1)	(0.143, 1.448, 4)	(3, 4, 5)
A2	(0.25, 4.083, 7)	(1, 1, 1)	(1.25, 5.75, 8)
A3	(0.2, 0.261, 0.333)	(0.125, 0.35, 0.8)	(1, 1, 1)

**Table 7 tab7:** Fuzzy pairwise comparison matrix for the clinical risk factors A4–A8 in Mild OSA in terms of TFN.

Factors	A4	A5	A6	A7	A8
A4	(1, 1, 1)	(0.25, 1.417, 3)	(0.5, 1.83, 4)	(0.2, 2.07, 3)	(0.14, 0.77, 2)
A5	(0.333, 1.778, 4)	(1, 1, 1)	(0.5, 1.33, 3)	(0.2, 1.07, 2)	(0.14, 1.44, 4)
A6	(0.25, 1.083, 2)	(0.333, 1.444, 2)	(1, 1, 1)	(0.25, 1.25, 3)	(0.14, 0.78, 2)
A7	(0.333, 1.889, 5)	(0.5, 2.167, 5)	(0.333, 2.11, 4)	(1, 1, 1)	(0.17, 0.81, 2)
A8	(0.5, 4.5, 7)	(0.25, 4.417, 7)	(0.5, 4.167, 7)	(0.5, 3.5, 6)	(1, 1, 1)

**Table 8 tab8:** Fuzzy pairwise comparison matrix for the clinical risk factors A9–A13 in Moderate OSA in terms of TFN.

Factors	A9	A10	A11	A12	A13
A9	(1, 1, 1)	(0.2, 1.733, 4)	(3, 4.667, 6)	(0.2, 0.233, 0.25)	(1, 2.333, 3)
A10	(0.25, 2.083, 5)	(1, 1, 1)	(2, 4.667, 8)	(0.14, 0.325, 0.5)	(0.333, 2.444, 5)
A11	(0.167, 0.233, 0.333)	(0.125, 0, 292, 0.5)	(1, 1, 1)	(0.14, 0.151, 0.167)	(0.333, 0.889, 2)
A12	(4, 4.333, 5)	(2, 4, 7)	(6, 6.667, 7)	(1, 1, 1)	(5, 6.667, 8)
A13	(0.333, 0.556, 1)	(0.2, 1.233, 3)	(0.5, 2.167, 3)	(0.13, 0.156, 0.2)	(1, 1, 1)

**Table 9:** Fuzzy pairwise comparison matrix for the clinical risk factors A14–A19 in Severe OSA in terms of TFN.

	A14	A15	A16	A17	A18	A19
A14	(1, 1, 1)	(0.25, 2.417, 4)	(0.14, 0.225, 0.333)	(0.33, 1.444, 2)	(0.333, 1.611, 4)	(0.143, 0.17, 0.2)
A15	(0.25, 1.528, 4)	(1, 1, 1)	(0.25, 0.333, 0.5)	(0.428, 1.643, 4)	(0.2, 1.567, 4)	(0.143, 0.181, 0.2)
A16	(3, 5, 7)	(2, 3.333, 4)	(1, 1, 1)	(2, 3.333, 4)	(0.5, 3.167, 6)	(0.2, 0.4, 0.5)
A17	(0.5, 1.333, 3)	(0.25, 1.528, 2.333)	(0.25, 0.333, 0.5)	(1, 1, 1)	(1, 2, 3)	(0.143, 0.225, 0.333)
A18	(0.25, 1.75, 3)	(0.25, 2.417, 5)	(0.17, 0.833, 2)	(0.333, 0.611, 1)	(1, 1, 1)	(0.143, 0.281, 0.5)
A19	(5, 6, 7)	(5, 6, 7)	(2, 3, 5)	(3, 5, 7)	(2, 4.667, 7)	(1, 1, 1)

**Table 10:** Fuzzy criteria weights of TFN in No OSA and OSA group.

Criteria	Decision factors	Weights of TFN
No OSA	*W*_A1_ = *S*_A1_	(0.149, 0.342, 1.26)
	*W*_A2_ = *S*_A2_	(0.09, 0.574, 2.016)
	*W*_A3_ = *S*_A3_	(0.048, 0.085, 0.269)

Mild OSA	*W*_A4_ = *S*_A4_	(0.025, 0.156, 1.144)
	*W*_A5_ = *S*_A5_	(0.026, 0.146, 1.232)
	*W*_A6_ = *S*_A6_	(0.024, 0.122, 0.88)
	*W*_A7_ = *S*_A7_	(0.028, 0.175, 1.496)
	*W*_A8_ = *S*_A8_	(0.033, 0.387, 2.464)

Moderate OSA	*W*_A9_ = *S*_A9_	(0.076, 0.199, 0.456)
	*W*_A10_ = *S*_A10_	(0.052, 0.21, 0.624)
	*W*_A11_ = *S*_A11_	(0.025, 0.05, 0.128)
	*W*_A12_ = *S*_A12_	(0.252, 0.453, 0.896)
	*W*_A13_ = *S*_A13_	(0.03, 0.102, 0.262)

Severe OSA	*W*_A14_ = *S*_A14_	(0.022, 0.103, 0.311)
	*W*_A15_ = *S*_A15_	(0.023, 0.094, 0.37)
	*W*_A16_ = *S*_A16_	(0.087, 0.243, 0.608)
	*W*_A17_ = *S*_A17_	(0.031, 0.096, 0.274)
	*W*_A18_ = *S*_A18_	(0.021, 0.099, 0.338)
	*W*_A19_ = *S*_A19_	(0.18, 0.385, 0.918)

**Table 11:** Degree of possibility of *V*(*S*_*j*_ ≥ *S*_*i*_) in No OSA.

*V*(*S*_A1_ ≥ *S*_*i*_)	Value	*V*(*S*_A2_ ≥ *S*_*i*_)	Value	*V*(*S*_A3_ ≥ *S*_*i*_)	Value

*V*(*S*_A1_ ≥ *S*_A2_)	0.835	*V*(*S*_A2_ ≥ *S*_A1_)	1	*V*(*S*_A3_ ≥ *S*_A1_)	0.318

*V*(*S*_A1_ ≥ *S*_A3_)	1	*V*(*S*_A2_ ≥ *S*_A3_)	1	*V*(*S*_A3_ ≥ *S*_A2_)	0.268

**Table 12 tab12:** Degree of possibility of *V*(*S*_*j*_ ≥ *S*_*i*_) in Mild OSA.

*V*(*S*_A4_ ≥ *S*_*i*_)	Value	*V*(*S*_*A*5_ ≥ *S*_*i*_)	Value	*V*(*S*_A6_ ≥ *S*_*i*_)	Value	*V*(*S*_A7_ ≥ *S*_*i*_)	Value	*V*(*S*_A8_ ≥ *S*_*i*_)	Value

*V*(*S*_A4_ ≥ *S*_A5_)	1	*V*(*S*_A5_ ≥ *S*_A4_)	0.992	*V*(*S*_A6_ ≥ *S*_A4_)	0.962	*V*(*S*_A7_ ≥ *S*_A4_)	1	*V*(*S*_A8_ ≥ *S*_A4_)	1

*V*(*S*_A4_ ≥ *S*_A6_)	1	*V*(*S*_A5_ ≥ *S*_A6_)	1	*V*(*S*_A6_ ≥ *S*_A5_)	0.973	*V*(*S*_A7_ ≥ *S*_A5_)	1	*V*(*S*_A8_ ≥ *S*_A5_)	1

*V*(*S*_A4_ ≥ *S*_A7_)	0.983	*V*(*S*_A5_ ≥ *S*_A7_)	0.976	*V*(*S*_A6_ ≥ *S*_A7_)	0.941	*V*(*S*_A7_ ≥ *S*_A6_)	1	*V*(*S*_A8_ ≥ *S*_A6_)	1

*V*(*S*_A4_ ≥ *S*_A8_)	0.828	*V*(*S*_A5_ ≥ *S*_A8_)	0.833	*V*(*S*_A6_ ≥ *S*_A8_)	0.762	*V*(*S*_A7_ ≥ *S*_A8_)	0.873	*V*(*S*_A8_ ≥ *S*_A7_)	1

**Table 13 tab13:** Degree of possibility of *V*(*S*_*j*_ ≥ *S*_*i*_) in Moderate OSA.

*V*(*S*_A9_ ≥ *S*_*i*_)	Value	*V*(*S*_A10_ ≥ *S*_*i*_)	Value	*V*(*S*_A11_ ≥ *S*_*i*_)	Value	*V*(*S*_A12_ ≥ *S*_*i*_)	Value	*V*(*S*_A13_ ≥ *S*_*i*_)	Value

*V*(*S*_A9_ ≥ *S*_A10_)	0.973	*V*(*S*_A10_ ≥ *S*_A9_)	1	*V*(*S*_A11_ ≥ *S*_A9_)	0.259	*V*(*S*_A12_ ≥ *S*_A9_)	1	*V*(*S*_A13_ ≥ *S*_A9_)	0.657

*V*(*S*_A9_ ≥ *S*_A11_)	1	*V*(*S*_A10_ ≥ *S*_A11_)	1	*V*(*S*_A11_ ≥ *S*_A10_)	0.322	*V*(*S*_A12_ ≥ *S*_A10_)	1	*V*(*S*_A13_ ≥ *S*_A10_)	0.66

*V*(*S*_A9_ ≥ *S*_A12_)	0.445	*V*(*S*_A10_ ≥ *S*_A12_)	0.605	*V*(*S*_A11_ ≥ *S*_A12_)	0	*V*(*S*_A12_ ≥ *S*_A11_)	1	*V*(*S*_A13_ ≥ *S*_A11_)	1

*V*(*S*_A9_ ≥ *S*_A13_)	1	*V*(*S*_A10_ ≥ *S*_A13_)	1	*V*(*S*_A11_ ≥ *S*_A13_)	0.65	*V*(*S*_A12_ ≥ *S*_A13_)	1	*V*(*S*_A13_ ≥ *S*_A12_)	0.028

**Table 14:** Degree of possibility of *V*(*S*_*j*_ ≥ *S*_*i*_) in Severe OSA.

*V*(*S*_A14_ ≥ *S*_*i*_)	Value	*V*(*S*_A15_ ≥ *S*_*i*_)	Value	*V*(*S*_A16_ ≥ *S*_*i*_)	Value	*V*(*S*_A17_ ≥ *S*_*i*_)	Value	*V*(*S*_A18_ ≥ *S*_*i*_)	Value	*V*(*S*_A19_ ≥ *S*_*i*_)	Value
*V*(*S*_A14_ ≥ *S*_A15_)	1	*V*(*S*_A15_ ≥ *S*_A14_)	0.975	*V*(*S*_A16_ ≥ *S*_A14_)	1	*V*(*S*_A17_ ≥ *S*_A14_)	0.973	*V*(*S*_A18_ ≥ *S*_A14_)	0.988	*V*(*S*_A19_ ≥ *S*_A14_)	1

*V*(*S*_A14_ ≥ *S*_A16_)	0.615	*V*(*S*_A15_ ≥ *S*_A16_)	0.655	*V*(*S*_A16_ ≥ *S*_A15_)	1	*V*(*S*_A17_ ≥ *S*_A15_)	1	*V*(*S*_A18_ ≥ *S*_A15_)	1	*V*(*S*_A19_ ≥ *S*_A15_)	1

*V*(*S*_A14_ ≥ *S*_A17_)	1	*V*(*S*_A15_ ≥ *S*_A17_)	0.994	*V*(*S*_A16_ ≥ *S*_A17_)	1	*V*(*S*_A17_ ≥ *S*_A16_)	0.56	*V*(*S*_A18_ ≥ *S*_A16_)	0.64	*V*(*S*_A19_ ≥ *S*_A16_)	1

*V*(*S*_A14_ ≥ *S*_A18_)	1	*V*(*S*_A15_ ≥ *S*_A18_)	0.986	*V*(*S*_A16_ ≥ *S*_A18_)	1	*V*(*S*_A17_ ≥ *S*_A18_)	0.988	*V*(*S*_A18_ ≥ *S*_A17_)	1	*V*(*S*_A19_ ≥ *S*_A17_)	1

*V*(*S*_A14_ ≥ *S*_A19_)	0.317	*V*(*S*_A15_ ≥ *S*_A19_)	0.395	*V*(*S*_A16_ ≥ *S*_A19_)	0.751	*V*(*S*_A17_ ≥ *S*_A19_)	0.245	*V*(*S*_A18_ ≥ *S*_A19_)	0.356	*V*(*S*_A19_ ≥ *S*_A18_)	1

**Table 15:** Minimum degree of possibilities value of each clinical risk factor in No OSA to Severe OSA.

Criteria	Minimum degree of possibilities values
No OSA	*d*′(*S*_A1_) = min⁡*V*(*S*_A1_ ≥ *S*_A2_, *S*_A3_) = min⁡(0.835,1) = 0.835
	*d*′(*S*_A2_) = min⁡*V*(*S*_A2_ ≥ *S*_A1_, *S*_A3_) = min⁡(1,1) = 1
	*d*′(*S*_A3_) = min⁡*V*(*S*_A3_ ≥ *S*_A1_, *S*_A2_) = min⁡(0.318,0.268) = 0.268

Mild OSA	*d*′(*S*_A4_) = min⁡*V*(*S*_A4_ ≥ *S*_A5_, *S*_A6_, *S*_A7_, *S*_A8_) = min⁡(1,1, 0.983,0.828) = 0.828
	*d*′(*S*_A5_) = min⁡*V*(*S*_A5_ ≥ *S*_A4_, *S*_A6_, *S*_A7_, *S*_A8_) = min⁡(0.992,1, 0.976,0.833) = 0.833
	*d*′(*S*_A6_) = min⁡*V*(*S*_A6_ ≥ *S*_A4_, *S*_A5_, *S*_A7_, *S*_A8_) = min⁡(0.962,0.973,0.941,0.762) = 0.762
	*d*′(*S*_A7_) = min⁡*V*(*S*_A7_ ≥ *S*_A4_, *S*_A5_, *S*_A6_, *S*_A8_) = min⁡(1,1, 1,0.873) = 0.873
	*d*′(*S*_A8_) = min⁡*V*(*S*_A8_ ≥ *S*_A4_, *S*_A5_, *S*_A6_, *S*_A7_) = min⁡(1,1, 1,1) = 1

Moderate OSA	*d*′(*S*_A9_) = min⁡*V*(*S*_A9_ ≥ *S*_A10_, *S*_A11_, *S*_A12_, *S*_A13_) = min⁡(0.973,1, 0.445,1) = 0.445
	*d*′(*S*_A10_) = min⁡*V*(*S*_A10_ ≥ *S*_A9_, *S*_A11_, *S*_A12_, *S*_A13_) = min⁡(1,1, 0.605,1) = 0.605
	*d*′(*S*_A11_) = min⁡*V*(*S*_A11_ ≥ *S*_A9_, *S*_A10_, *S*_A12_, *S*_A13_) = min⁡(0.259,0.322,0, 0.65) = 0
	*d*′(*S*_A12_) = min⁡*V*(*S*_A12_ ≥ *S*_A9_, *S*_A10_, *S*_A11_, *S*_A13_) = min⁡(1,1, 1,1) = 1
	*d*′(*S*_A13_) = min⁡*V*(*S*_A13_ ≥ *S*_A9_, *S*_A10_, *S*_A11_, *S*_A12_) = min⁡(0.657,0.66,1, 0.028) = 0.028

Severe OSA	*d*′(*S*_A14_) = min⁡*V*(*S*_A14_ ≥ *S*_A15_, *S*_A16_, *S*_A17_, *S*_A18_, *S*_A19_) = min⁡(1,0.615,1, 1,0.317) = 0.317
	*d*′(*S*_A15_) = min⁡*V*(*S*_A15_ ≥ *S*_A14_, *S*_A16_, *S*_A17_, *S*_A18_, *S*_A19_) = min⁡(0.975,0.655,0.994,0.986,0.395) = 0.395
	*d*′(*S*_A16_) = min⁡*V*(*S*_A16_ ≥ *S*_A14_, *S*_A15_, *S*_A17_, *S*_A18_, *S*_A19_) = min⁡(1,1, 1,1, 0.751) = 0.751
	*d*′(*S*_A17_) = min⁡*V*(*S*_A17_ ≥ *S*_A14_, *S*_A15_, *S*_A16_, *S*_A18_, *S*_A19_) = min⁡(0.973,1, 0.56,0.988,0.245) = 0.245
	*d*′(*S*_A18_) = min⁡*V*(*S*_A18_ ≥ *S*_A14_, *S*_A15_, *S*_A16_, *S*_A17_, *S*_A19_) = min⁡(0.988,1, 0.64,1, 0.356) = 0.356
	*d*′(*S*_A19_) = min⁡*V*(*S*_A19_ ≥ *S*_A14_, *S*_A15_, *S*_A16_, *S*_A17_, *S*_A18_) = min⁡(1,1, 1,1, 1) = 1

**Table 16:** Weight vector (*w*′) and normalized weight vector (*w*) of each clinical risk factor.

Criteria	Weight vector (*w*′)	Normalized weight vector (*w*)
No OSA	(0.835, 1, 0.268)	(0.397, 0.476, 0.127)
Mild OSA	(0.828, 0.833, 0.762, 0.873, 1)	(0.193, 0.194, 0.177, 0.203, 0.233)
Moderate OSA	(0.445, 0.605, 0, 1, 0.028)	(0.214, 0.291, 0, 0.481, 0.013)
Severe OSA	(0.317, 0.395, 0.751, 0.245, 0.356, 1)	(0.103, 0.129, 0.245, 0.08, 0.116, 0.326)

**Table 17:** Normalized weight vector of each clinical risk factor, its ranking in OSA, and sensitivity/specificity with 95% CI of each severity group.

Criteria	Factors	Normalized weight vector	Ranking	Sensitivity% (95% CI)
				Specificity% (95% CI)
Severe OSA	Witnessed snoring and apnea (A19)	0.326	1	92.32 89.62–94.51
	Symptoms at nighttime awakening (A16)	0.245	2	
	Body built (A15)	0.129	3	
	Sleep maintenance (A18)	0.116	4	91.76 88.82–94.13
	Epworth Sleepiness Scale (7/8) (A14)	0.103	5	
	Underlying diseases and personal variables (A17)	0.08	6	

Moderate OSA	Witnessed snoring and apnea plus awakening due to chest discomfort (A12)	0.481	1	89.52 84.57–93.32
	Decreased mental and physical performance (A10)	0.291	2	
	Epworth Sleepiness Scale (6/8) (A9)	0.214	3	88.18 85.51–90.52
	Underlying diseases and surreal dream (A13)	0.013	4	
	Related personal variables (A11)	0	5	

Mild OSA	Choking and witnessed snoring (A8)	0.233	1	91.08 85.49–95.04
	Risk variables and work (A7)	0.203	2	
	Epworth Sleepiness Scale (3/8) (A5)	0.194	3	84.58 81.65–87.21
	Causes of nighttime awakening (A4)	0.193	4	
	Lung diseases and sleep-wake pattern (A6)	0.177	5	

No OSA	Symptoms during sleep (A2)	0.476	1	96.49 87.89–99.57
	Epworth Sleepiness Scale (2/8) and related variables (A1)	0.397	2	
	Underlying diseases and sleep posture (A3)	0.127	3	81.23 78.31–83.92

**Table 18:** The clinical risk factors and variables for urgent sleep study appointment.

Clinical risk factors	Variables	Sensitivity% (95% CI)	Specificity% (95% CI)
(1) Witnessed snoring and apnea	Snoring		
	Loudness of your snoring		
	Periodically stopped breathing		
(2) Symptoms at nighttime awakening	Difficult breathing like something obstructive in the throat		
	Feeling like choking		
	Leg or arm jerking		
(3) Body built	Waist circumference Hip circumference Body mass index Thyromental distance		
(4) Sleep maintenance	Urinating very often Getting up earlier than your expectation Waking up during late night (times/night)	92.32 89.62–94.51	91.76 88.82–94.13
(5) Epworth Sleepiness Scale (7/8)	Sitting inactive in a public place Sitting quietly after lunch with no alcohol Being in a car for an hour without break Lying down to rest in the afternoon Sitting and reading Watching TV Sitting and talking to someone		
(6) Underlying diseases and personal variables	Hypertension Routine medicine use Age Diabetes mellitus		

**Table 19:** The clinical risk factor and variables in screening for Mild from No OSA in sleep center setting.

Clinical risk factor	Variables	Sensitivity% (95% CI)	Specificity% (95% CI)
Symptoms during sleep	Periodically grasping hands	87.12 80.98–91.84	72.22 58.36–83.54
	Chest discomfort		
	Sweating profoundly during sleep		
